# Outcome indicators and effectiveness of nutrition education for athletes: a systematic review

**DOI:** 10.1186/s13102-026-01900-9

**Published:** 2026-07-21

**Authors:** Wakako Tatsuta, Saki Nakamura, Takayo Inayama, Emi Nakazaki, Mutsumi Ogawa

**Affiliations:** 1 College of Sports Sciences, Nihon University, 3-34-1 Shimoma, Setagaya-Ku, Tokyo, 154-8513 Japan; 2 Graduate Program in Sciences and Culture, Showa Women’s University, 1-7-57 Taishido, Setagaya-Ku, Tokyo, 154-8533 Japan; 3 Department of Nutrition, Chiba Prefectural University of Health Sciences, 2-10-1 Wakaba, Mihama-Ku, Chiba, 261-0014 Japan; 4 Department of Food and Health Sciences, Faculty of Health and Human Development, The University of Nagano, 8-49-7 Miwa, Nagano, 380-8525 Japan; 5 Hokuriku Institute of Wellness and Sports, Ishikawa, i13-1 Yawata, Komatsu, Ishikawa 923-8601 Japan; 6 Former affiliation: Graduate School of Health and Nutrition Sciences, The University of Nagano, 8-49-7 Miwa, Nagano, 380-8525 Japan

**Keywords:** Athletes, Nutrition education, Health and nutritional status, Outcome indicators, Systematic review

## Abstract

**Background:**

Appropriate management of health and nutritional status is essential for athletes to maintain performance, enhance recovery, and prevent injuries. Nutrition education aims to improve health and nutritional status through changes in dietary behaviors, thereby supporting athletic sustainability and quality of life. Previous systematic reviews have primarily focused on intermediate outcomes such as nutrition knowledge and behaviors, whereas health and nutritional status as final outcomes and the effectiveness of nutrition education interventions have not been systematically organized. This review aimed to (1) summarize health and nutritional status outcome measures used in nutrition education intervention studies targeting athletes and (2) examine the effectiveness of these interventions in relation to outcome consistency and alignment with intervention objectives.

**Methods:**

This systematic review was conducted in accordance with PRISMA guidelines and registered in PROSPERO (CRD42023417419). Seven databases were searched for studies published between April 2003 and March 2025. Eligible studies included randomized controlled trials, non-randomized controlled trials, and pre–post intervention studies involving athletes aged ≥ 12 years. Nutrition education interventions included lectures, counseling, and face-to-face or online programs delivered to individuals and/or groups. Outcomes included objective and/or subjective indicators of health and nutritional status.

**Results:**

Twenty-four studies involving 845 athletes were included. All studies assessed anthropometric or body composition measures such as body weight, fat mass, and fat-free mass. Biochemical indicators were less frequently used, with hydration status assessed by urine specific gravity in three studies and blood biomarkers in three studies. Subjective health-related outcomes were rarely evaluated. Intermediate outcomes such as nutrition knowledge and dietary behaviors were assessed in 12 studies. Studies in which intervention objectives, educational content, and outcome measures were aligned tended to report improvements corresponding to their stated objectives.

**Conclusions:**

In nutrition education intervention studies targeting athletes, objective measures of body weight and body composition are commonly used, whereas subjective health status assessments remain limited. Nutrition education may be effective for intermediate outcomes such as nutrition knowledge and dietary behaviors, but evidence for effectiveness in physiological and biochemical outcomes remains inconclusive. Aligning intervention objectives, educational content, and evaluation measures within a nutrition management framework may be important for appropriately evaluating effectiveness. Repeated nutrition education interventions may contribute to sustainable behavioral and health-related changes in athletes.

**Supplementary Information:**

The online version contains supplementary material available at https://doi.org/10.1186/s13102-026-01900-9.

## Background

Appropriate management of health and nutritional status in athletes is essential for maintaining and enhancing performance and for preventing injuries [1, 2]. High-intensity and high-frequency training imposes substantial physical demands, and inadequate nutrient intake may delay recovery from fatigue and increase the risk of injury. Indeed, nutritional challenges such as low energy availability and iron deficiency have been reported among many athletes [3, 4], emphasizing the importance of nutrition education in sports settings [5].

Nutrition education is an intervention aimed at maintaining and improving health and nutritional status through changes in dietary behaviors, ultimately contributing to athletes’ quality of life (QOL) and the sustainability of athletic participation [6, 7]. Several systematic reviews have examined nutrition education interventions targeting athletes. Tam et al. demonstrated that nutrition education interventions are effective in improving nutrition knowledge [8], while Sánchez-Díaz reported that nutrition education interventions for team sport athletes contribute to improvements in knowledge and behavioral change [9]. In contrast, Bentley et al. pointed out that nutrition education interventions for athletes incorporating health behavior theories and behavior change techniques are limited [10], and Boidin et al. reported that evidence regarding the effectiveness of nutrition education in improving dietary intake remains inconclusive [11]. In addition, Janiczak et al. reported that interventions targeting modifiable factors influencing dietary intake in athletes demonstrated mixed effectiveness, highlighting the importance of behavioral and environmental factors [12].

These reviews have primarily focused on intermediate outcomes such as nutrition knowledge and dietary behaviors and have not systematically organized health and nutritional status itself as outcome indicators. Although nutrition education is expected to lead to improvements in health and nutritional status and QOL through changes in dietary behavior, the specific health and nutritional status outcome indicators used in nutrition education interventions for athletes have not been sufficiently summarized. The International Olympic Committee (IOC) international nutrition guidelines for athletes emphasize the use of objective health-related indicators for outcome evaluation [13]; however, the extent to which nutrition education interventions are effective in improving these indicators remains unclear.

Furthermore, the effectiveness of nutrition education interventions should be interpreted in relation to the degree of alignment among intervention objectives, educational content, and outcome measures, as well as the relationship between intermediate outcomes, such as knowledge and behaviors, and changes in health and nutritional status. However, to date, no review has comprehensively examined nutrition education intervention studies for athletes from these perspectives.

Therefore, it is necessary to organize appropriate health and nutritional status outcome indicators used as final outcomes in nutrition education interventions for athletes and to clarify the characteristics of interventions that are effective in improving these indicators. This is also important for systematizing nutrition education practices for athletes based on scientific evidence.

The objectives of this systematic review were as follows: (1) to summarize health and nutritional status outcome indicators assessed before and after nutrition education interventions targeting athletes; and (2) to examine the effectiveness of these interventions in relation to the consistency of outcome changes and the relationships among intervention objectives, educational content, and intermediate outcomes.

## Methods

### Study design and registration

This systematic review was conducted and reported in accordance with the PRISMA statement [14] and registered in PROSPERO (CRD42023417419).

A systematic literature search was conducted in seven databases—PubMed, Web of Science, SPORTDiscus, Cochrane Library, ERIC, Embase, and ScienceDirect—for studies published between April 1, 2003, and March 31, 2023. The year 2003 was selected as the starting point because it corresponds to the period when international sports nutrition guidelines, such as the IOC Nutrition for Athletes, began to be established.

The search strategy combined the following keywords:

“athletes” AND (“education intervention” OR “nutritional intervention” OR “nutrition education” OR “diet education” OR “nutritional support” OR “nutritional program”) AND (“health status” OR “nutritional status” OR “nutrition assessment” OR “condition” OR “performance”).

The search strategy was developed by two authors (TW and IT), and the systematic search was conducted by one author (TW) in 2023.

To include newly published studies, the literature search was updated on March 31, 2025. The updated search used the same seven databases, search terms, and inclusion and exclusion criteria as the original search and covered studies published between April 1, 2023, and March 31, 2025. Newly identified studies were screened, and data extraction and quality assessment were performed using the same procedures as in the initial search.

### Eligibility criteria

Eligible participants were athletes aged 12 years or older, with no restrictions on sport type, discipline, or competitive level. Eligible study designs included randomized controlled trials (RCTs), non-randomized controlled trials (NRCTs), and single-group pre–post intervention studies.

Nutrition education interventions were delivered to individuals and/or groups and included lectures, counseling, and face-to-face and/or online formats. No restrictions were placed on intervention duration. Nutrition education was defined as an intervention aimed at improving dietary behaviors through information provision and behavioral support.

Studies that required mandatory consumption of dietary supplements or prescribed meals were excluded. However, studies were included if both the intervention and control groups consumed the same foods while only the intervention group received nutrition education, as the effect of nutrition education could be evaluated under these conditions. Studies that provided partial meals during the intervention period were also included, provided that food consumption was not mandatory and was considered an educational medium rather than a compulsory dietary intervention.

Eligible outcomes included pre–post assessments of objective health and nutritional status indicators (e.g., body weight, BMI, body composition, and blood parameters) and subjective health-related indicators. Studies were considered eligible even if the evaluation of health or nutritional status was not explicitly stated as an objective, provided that these outcomes were reported in the Methods or Results sections. Only peer-reviewed articles published in English were considered eligible.

### Study selection and data extraction

After duplicate records were removed, two authors (TW and NE) independently screened all titles and abstracts. Different authors participated in different stages of the screening process to distribute the workload while maintaining independent assessment at each stage. Full-text screening of all studies that met the eligibility criteria was independently conducted by two authors (TW and NS), with disagreements resolved through discussion with a third author (IT).

Outcomes were classified based on the Knowledge–Attitude–Practice (KAP) model into intermediate outcomes (knowledge, attitudes, self-efficacy, and dietary behaviors) and final outcomes (body composition, blood biomarkers, hydration status, and subjective health status). Data extraction was performed by two authors (TW and NS).

The following data were extracted: study characteristics (authors and year of publication), participant characteristics (country, age, sex, sample size, sport type, and discipline), outcomes, and intervention characteristics (intervention objectives, learning format, duration and frequency, nutrition education content, and intervention providers). Descriptions of nutrition education and nutrition counseling formats were classified in accordance with previous research [15].

Study designs were categorized as RCTs, NRCTs, or single-group pre–post intervention studies. Studies described by the authors as RCTs or NRCTs were reclassified as single-group pre–post intervention studies if the reported results were limited to within-group pre–post comparisons. When intervention objectives were not explicitly stated, they were determined by the authors based on contextual information.

### Quality assessment

Study quality was evaluated from two perspectives: (1) methodological quality and (2) the structural quality of nutrition education programs.

Methodological quality was independently assessed by two authors (TW and NS). Based on previous reviews [8–11], the use of conventional risk-of-bias tools was considered; however, because many included studies employed single-group designs and because widely used rigorous assessment tools may not be fully applicable to nutrition education interventions in sports settings [16, 17], the National Heart, Lung, and Blood Institute (NHLBI) Study Quality Assessment Tools were selected [18]. The tool Quality Assessment of Controlled Intervention Studies was used for RCTs, and the Quality Assessment Tool for Before–After (Pre–Post) Studies With No Control Group was used for NRCTs and single-group pre–post intervention studies, consisting of 14 and 12 items, respectively.

The structural quality of nutrition education programs was evaluated using a 15-item checklist developed based on the GENIE framework (Guide for Effective Nutrition Interventions and Education) proposed by Hand et al. [19], with adaptations to ensure relevance to the context of sports nutrition education.

Each item was classified as “yes,” “no,” or “other” (CD, NR, NA). Disagreements between reviewers were resolved through discussion. To ensure transparency, explicit criteria were established in advance for each item of the structural quality checklist. Items were rated as “yes” when clear and sufficient information meeting the predefined criteria was reported, and as “no” when information was missing, unclear, or insufficient.

## Results

### Study selection

**Fig. 1:**
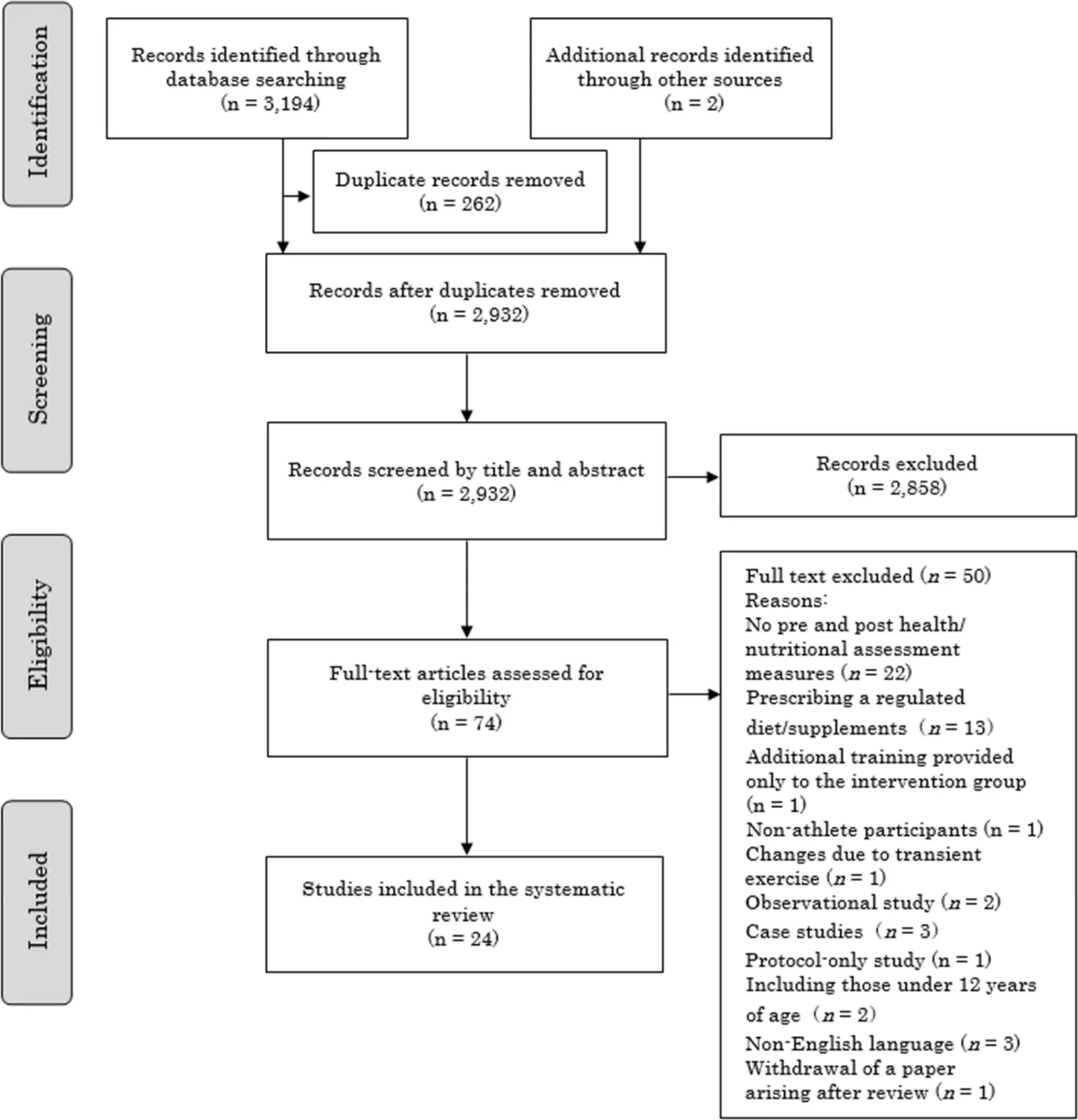
PRISMA flow chart showing the study selection process

Figure 1 presents an overview of the PRISMA flow diagram integrating the results of the initial search (April 1, 2003–March 31, 2023) and the updated search (April 1, 2023–March 31, 2025). A total of 3,194 records were identified through database searching. After removal of duplicates, 2,932 records were screened. Title and abstract screening identified 74 articles for full-text review. Of these, 50 articles were excluded, and 23 studies were included from the database search. Subsequently, one retracted article was excluded, and two additional studies were identified through manual reference searching, resulting in a final inclusion of 24 studies.

The characteristics of the included studies, participant characteristics, and intervention outcomes are summarized in Table 1.

**Table 1:**
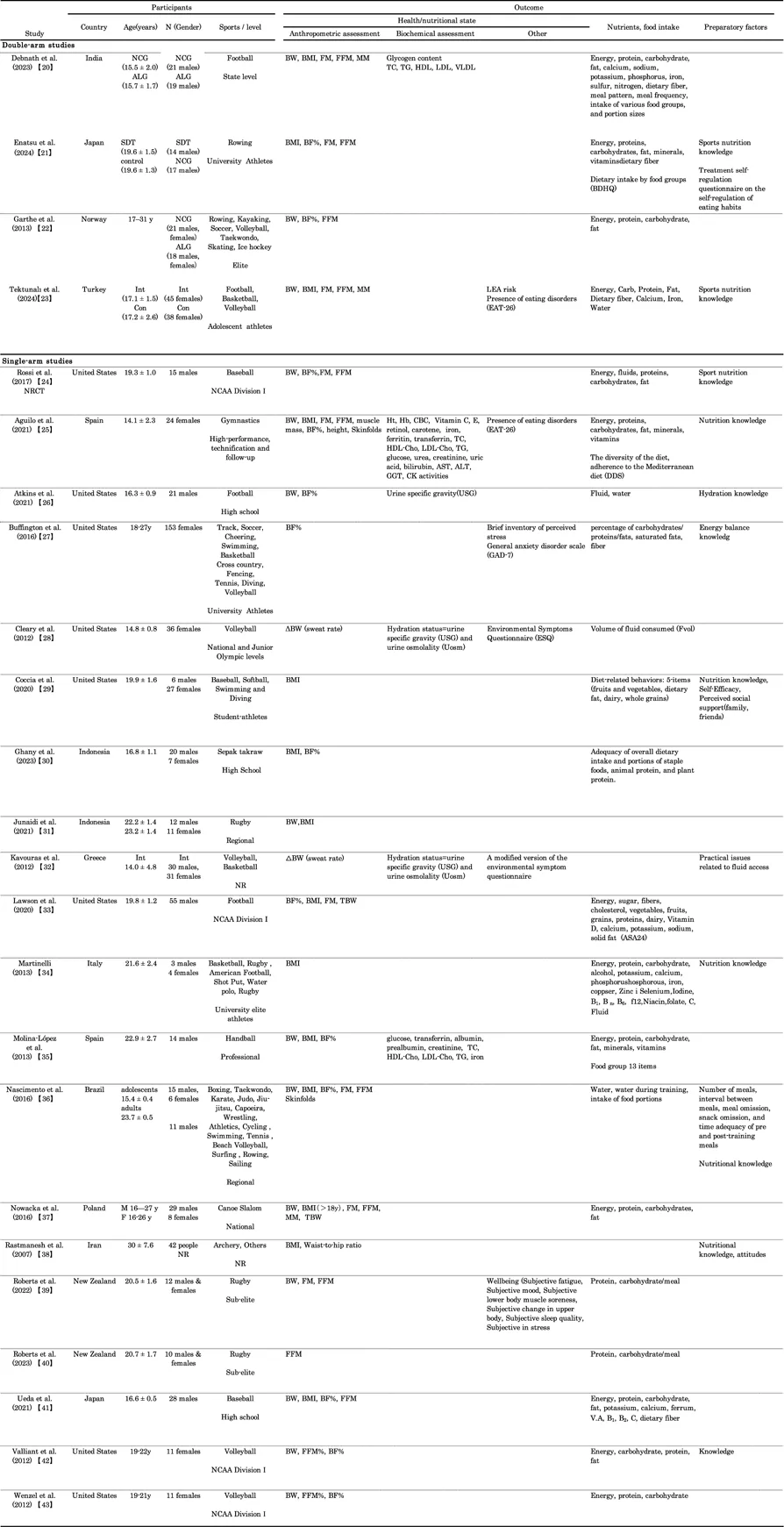
Characteristics of included studies and health- and nutrition-related outcomes [20–43]

### Participant characteristics

The included studies were conducted in North America (*n* = 8) [24, 26–29, 33, 42, 43], Europe (*n* = 7) [22, 23, 25, 32, 34, 35, 37], Asia (*n* = 6) [20, 21, 30, 31, 38, 41], Oceania (*n* = 2) [39, 40], and South America (*n* = 1) [36]. Sample sizes ranged from 7 to 153 participants. Among the total of 845 participants, 39% were male, 49% were female, and sex was not reported for 12%.

Sports disciplines varied widely. Sixteen studies focused on a single sport, including both individual and team sports [20, 21, 24–26, 28, 30, 31, 33, 35, 37, 39–43], while eight studies included participants from multiple sports or disciplines [22, 23, 27, 29, 32, 34, 36, 38]. Competitive levels included high school (*n* = 4) [23, 26, 30, 41], collegiate (*n* = 7) [21, 27, 29, 33, 34, 42, 43], regional level (*n* = 3) [20, 31, 36], national or elite level (*n* = 3) [22, 28, 37], and professional athletes (*n* = 1) [35]. One study included athletes with physical disabilities [38].

### Outcome characteristics

All 24 studies assessed anthropometric or body composition measures, including body weight. Objective physiological and biochemical indicators were also included, such as blood biomarkers related to anemia (*n* = 3) [20, 25, 35] and hydration status assessed by urine specific gravity (*n* = 3) [26, 28, 32]. In addition, subjective outcome measures were used, including the Eating Attitudes Test-26 (EAT-26) for screening eating disorders (*n* = 2) [23, 25], the Environmental Symptoms Questionnaire (ESQ) (*n* = 2) [28, 32], well-being (*n* = 1) [39], and indicators of stress and anxiety (*n* = 1) [27].

Twelve studies evaluated intermediate outcomes corresponding to the KAP model, such as nutrition knowledge, dietary behaviors, and self-efficacy [21, 23–27, 29, 32, 34, 36, 38, 42].

### Intervention characteristics and effectiveness

The characteristics of the interventions are summarized in Table 2. Intervention objectives included improvements in body weight or body composition [20, 22, 32, 40], dehydration prevention [26, 28, 32], improvement of lipid profiles [20], and reductions in eating disorder risk or low energy availability (LEA) risk [23]. Some studies set intervention objectives related to preparatory factors such as knowledge, behaviors, and self-efficacy, while other studies did not clearly describe intervention objectives.

**Table 2:**
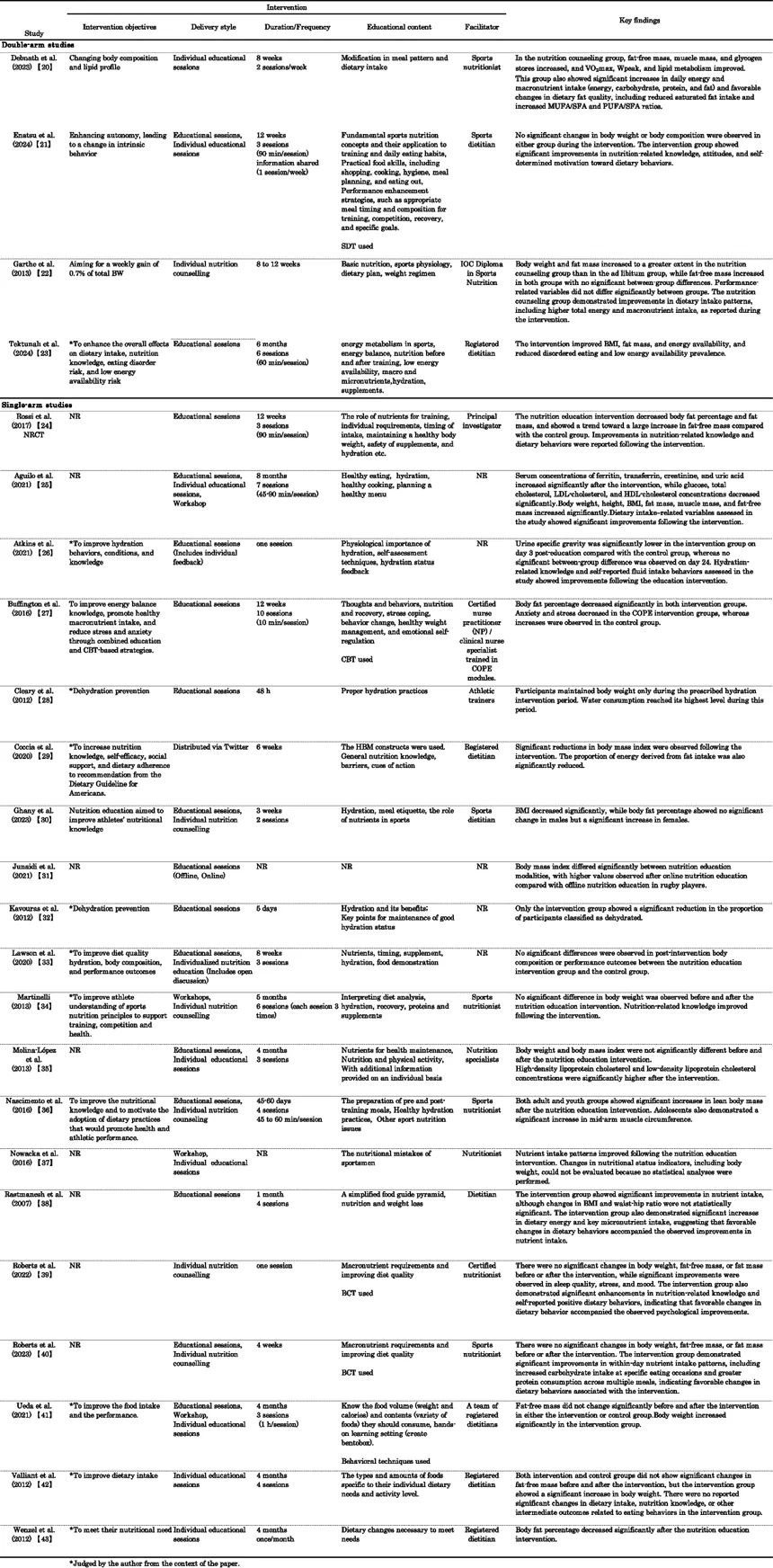
Characteristics and components of nutrition education interventions in athletes [20–43]

Learning formats and educational content varied, including face-to-face and online delivery, as well as individual and group-based formats. Several studies employed combinations of multiple formats and topics (Table 2). Educational content was broadly categorized into dietary intake and nutrients, hydration, timing of intake, supplements, weight management (weight gain or loss), and LEA. In most studies, interventions were delivered by registered dietitians, sports nutrition specialists, or nutrition researchers. Health behavior theories were explicitly reported in 6 of the 24 studies.

Fifteen studies demonstrated alignment between intervention objectives and educational content [20–23, 26–29, 32–34, 36, 40–42]. Among these, eight studies clearly specified health improvement as an outcome objective and implemented educational content consistent with that objective [20, 22, 23, 26, 28, 32, 33, 40]. All three studies aiming to prevent dehydration reported favorable outcomes [26, 28, 32]. In contrast, among studies aiming to promote weight gain or changes in body composition (*n* = 4) [20, 22, 33, 40], only two reported improvements [20, 22]. The study aiming to reduce eating disorder risk reported improvement [23].

Three studies explicitly stated health improvement as an outcome objective, implemented corresponding educational content, and evaluated intermediate outcomes [23, 26, 32]. Among these, two studies focusing on dehydration prevention demonstrated consistent improvements in both intermediate and final outcomes [26, 32].

### Quality assessment of study design

The results of the methodological quality assessment are presented in Tables 3 and 4. The mean score for RCTs was 8 out of 14 (range: 5–11). The mean score for NRCTs and single-group pre–post intervention studies was 5.6 out of 12 (range: 4–8). Although study objectives and outcome definitions were clearly described in many studies, reporting was often insufficient regarding population representativeness, sample size justification, blinding of interventions, and the use of individual-level data.

**Table 3:**
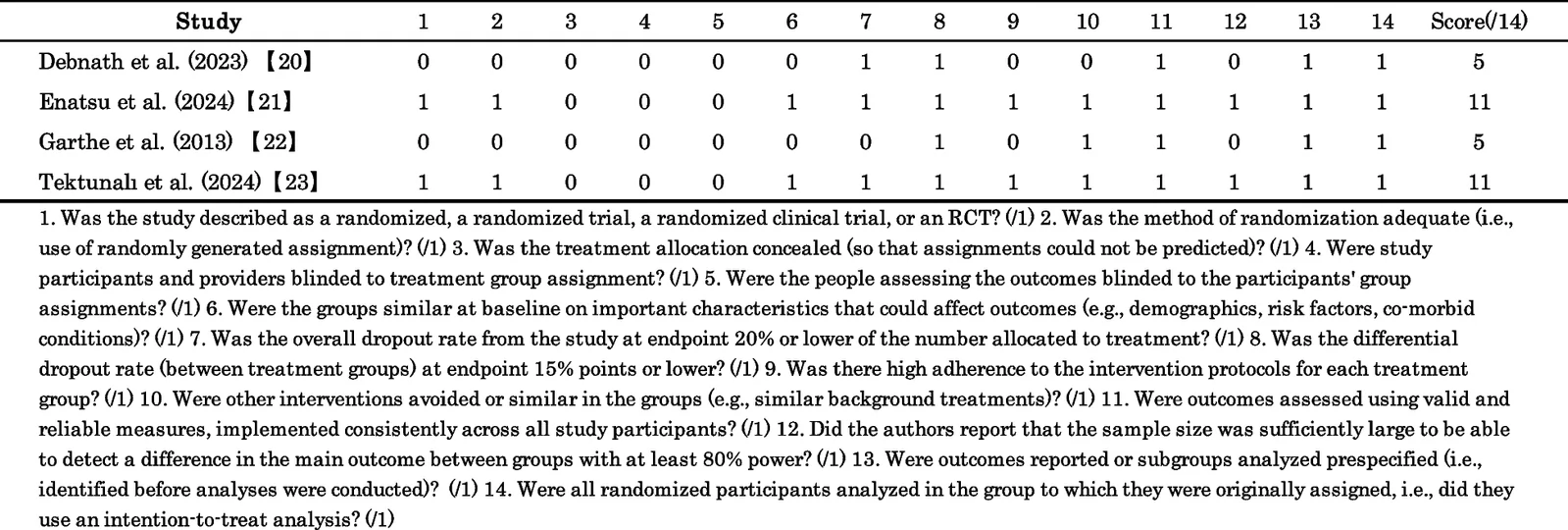
Methodologic quality of the included RCT [20–23]

**Table 4:**
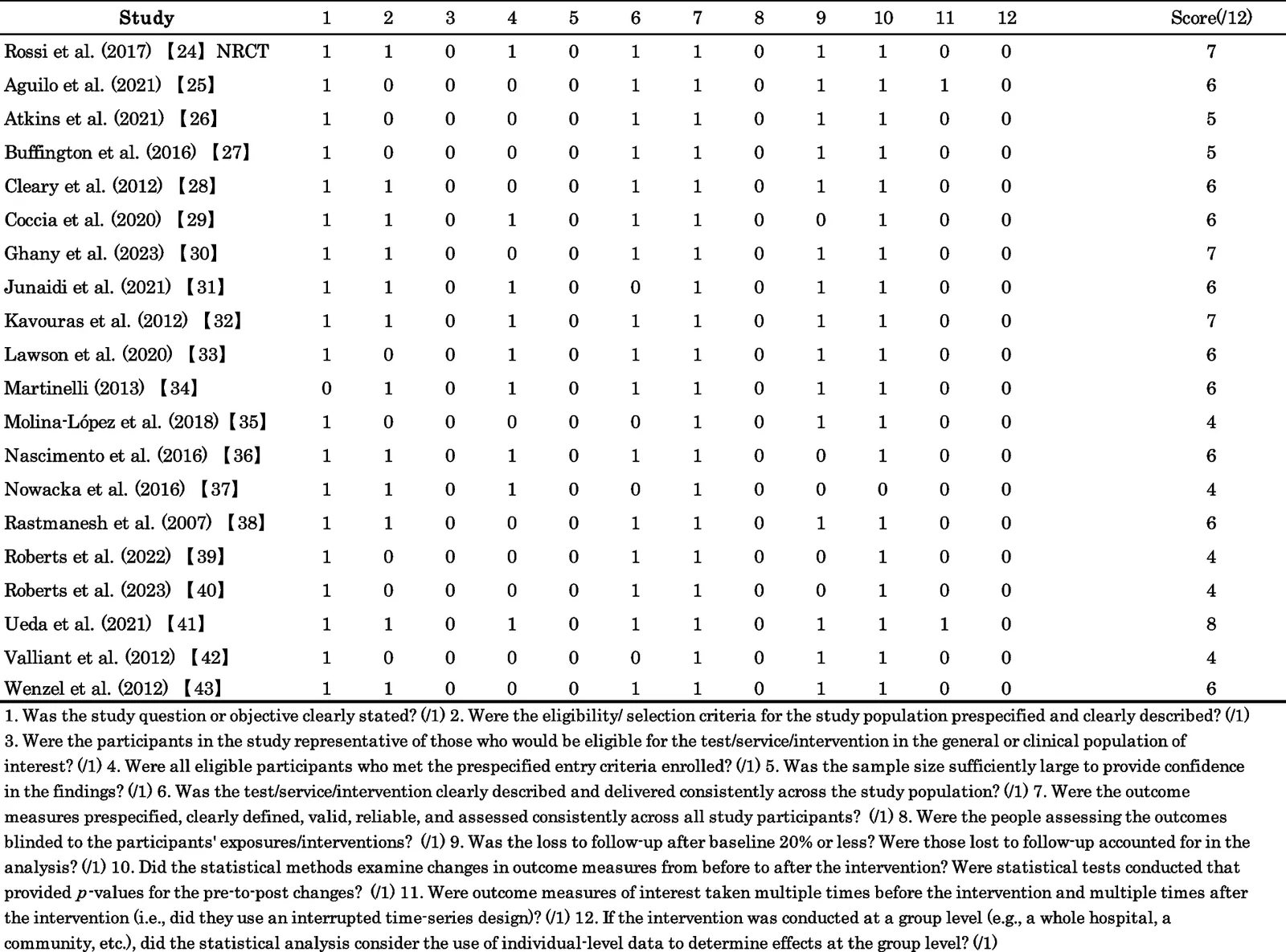
Methodologic quality of the included NRCT and single-arm [24–43]

The structural quality of the nutrition education programs is presented in Table 5. The mean score was 7 out of 15 (range: 3–10). Although some studies identified health-related issues of the target population based on previous research or general assumptions, no studies were found that clearly defined health issues based on pre-intervention assessments of the actual study participants.

**Table 5:**
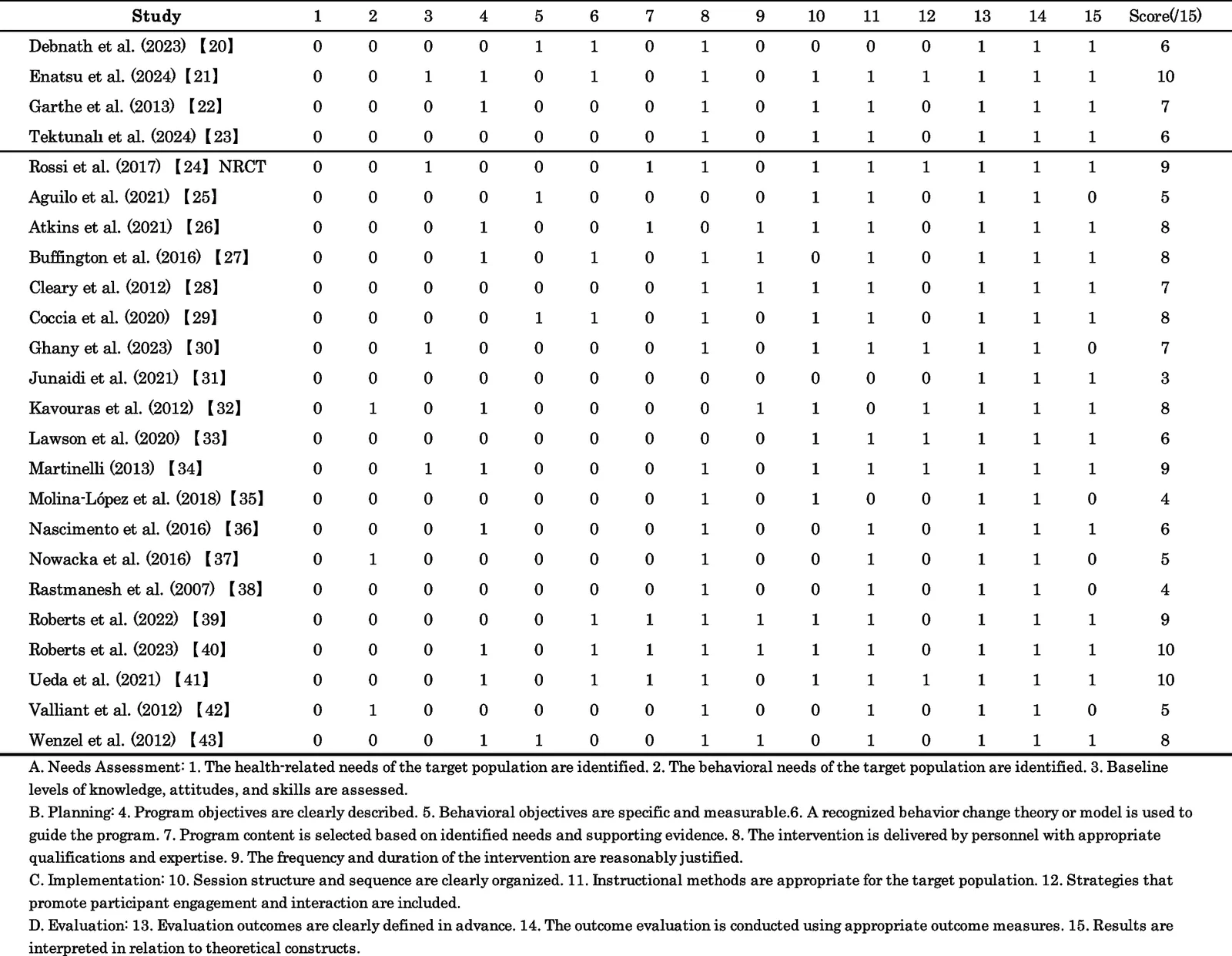
Structural quality assessment of nutrition education interventions based on the Guide for Effective Nutrition INterventions and Education (GENIE) [20–43]

## Discussion

This systematic review organized outcome indicators related to health and nutritional status (final outcomes) used in nutrition education intervention studies targeting athletes and examined their effectiveness in relation to the consistency of outcome changes and the alignment among intervention objectives, educational content, and intermediate outcomes. The results showed that anthropometric and body composition measures, such as body weight, fat mass (FM), and fat-free mass (FFM), were the most frequently used indicators to assess health and nutritional status. In contrast, the use of physiological and biochemical indicators, as well as subjective health status measures, was limited. Furthermore, among the 24 included studies, only six clearly defined outcome objectives related to health and nutritional status, implemented corresponding educational content, and reported improvements in those outcomes [20, 23, 26–28, 32]. These findings indicate that, given the historical focus of nutrition education research on intermediate outcomes, it is necessary to consider not only the appropriateness of outcome indicators but also the alignment among intervention objectives, educational content, and evaluation measures when examining the effectiveness of nutrition education interventions.

Previous studies have reported that nutrition education is effective in improving nutrition knowledge and dietary behaviors [8, 9]. Similar trends were observed in the present review; however, inconsistencies in changes in dietary intake, as reported by Boidin et al. [11], were also evident. Thus, this review extends existing literature by providing additional insights relevant to the design of nutrition education programs for athletes.

In this review, improvements in intermediate outcomes—specifically nutrition knowledge, dietary behaviors, and self-efficacy—were reported in many studies. These changes correspond to the early stages of cognitive and behavioral change within the KAP model. In particular, interventions incorporating behavioral science theories appeared to facilitate progression toward behavioral change. However, improvements in intermediate outcomes did not necessarily translate into improvements in final outcomes related to health and nutritional status. The relationship between intermediate and final outcomes appeared to depend on intervention objectives and the selection of outcome indicators.

In contrast, many educational interventions focusing primarily on the provision of nutrition knowledge did not demonstrate clear improvements in physiological or biochemical indicators, such as blood biomarkers. Based on the limited number of studies, consistent improvements in these indicators were not observed. One possible explanation is that improvements in intermediate outcomes, such as knowledge and behaviors, may not be reflected in physiological or biochemical changes over a short period [44, 45]. Accordingly, interventions and evaluations conducted from a mid- to long-term perspective may be necessary to adequately capture changes in health and nutritional status.

### Health- and nutrition-related outcome indicators used in nutrition education interventions

Body weight, an anthropometric or body composition measure, was used in all included studies. In addition, urine specific gravity was used as a physiological or biochemical indicator in all three studies that aimed to prevent dehydration. These objective indicators are non-invasive and practical measures that allow for simple assessment of energy balance [46] and hydration status [47]. Although blood tests provide important information on nutritional adequacy and deficiencies [48], they were used in only three studies. Compared with body weight or urine specific gravity measurements, the invasiveness and economic burden of blood testing may have limited their use. The use of subjective outcome measures was also limited. Although such measures are highly practical in nutrition education settings, few standardized and validated subjective health status scales for athletes are currently available. The development of such scales may facilitate evaluation, particularly in nutrition education programs targeting entire teams.

### Effectiveness of nutrition education interventions

Considering the characteristics of the outcome indicators used, the effectiveness of nutrition education interventions appeared to depend largely on intervention objectives, educational content, and the selection of outcome measures. In the present review, studies demonstrating effectiveness were characterized by clearly defined intervention objectives and corresponding educational content. All three studies that aimed to prevent dehydration reported improvements in urine specific gravity and related indicators, indicating effectiveness even in short-term interventions. Programs focusing on hydration and energy balance frequently reported improvements in objective indicators such as body weight and urine specific gravity. In these studies, intervention objectives, educational content, and outcome measures were generally well aligned, highlighting the importance of implementing nutrition education in accordance with nutrition management principles in athletic settings.

In contrast, studies aiming to improve body composition or promote weight gain showed inconsistent effects, despite intervention durations ranging from 1 day to 8 months**.** Furthermore, among the 24 included studies, only six clearly described intervention objectives that aligned with the selected outcome indicators. In nutrition education interventions targeting non-athlete populations, factors such as intervention duration, planning based on appropriate health behavior theories, and clearly defined goal setting have been identified as determinants of effectiveness [49, 50]. However, only three of the 24 included studies implemented long-term education programs lasting five months or longer [23, 25, 34]. In these longer-term studies, improvements in body composition and lipid profiles were reported. These findings suggest that continuous education delivered multiple times over a period of at least two months may facilitate the consolidation of behavioral change. Given that changes in health and nutritional status are likely to occur after behavioral changes have been established, sufficient intervention duration should be considered in future studies.

Health behavior theories, such as the Transtheoretical Model [51], have been emphasized as important components of many educational interventions [52–54]. However, a lack of health behavior theory has been noted in nutrition education interventions targeting athletes [10], and in the present review, only four studies explicitly reported the use of health behavior theories or behavior change techniques related to nutrition education implementation [29, 39–41]. Among these, three studies [39–41] demonstrated behavioral changes and improvements in nutrient intake that were associated with improvements in health and nutritional status. These findings suggest that incorporating appropriate health behavior theories may enhance the effectiveness of nutrition education interventions by facilitating behavioral change that leads to improvements in health and nutritional status.

With regard to learning formats, relatively favorable outcomes were reported in studies that combined multiple approaches, such as group lectures and individual counseling. In studies that implemented counseling, behavior change techniques such as individualized goal setting, self-monitoring, and motivational interviewing were used. These approaches are broadly consistent with the definition of “nutrition counseling” proposed by Fiorini et al. [15] and represent a behavioral support–oriented approach rather than clinical treatment. This suggests that supplementing group-based education with individualized support may enhance effectiveness. Programs delivered by registered dietitians or sports nutrition specialists tended to report more consistent improvements in objective indicators such as body composition and nutritional status. In contrast, interventions delivered by trainers or coaches alone reported limited outcomes, underscoring the importance of multidisciplinary collaboration involving nutrition professionals, coaches, and trainers.

Overall, to promote improvements in health and nutritional status through dietary behavior change, nutrition education in sports settings should be implemented within a nutrition management framework that aligns intervention objectives, educational content, and evaluation measures. In particular, the development of assessment tools capable of capturing athletes’ subjective health status from multiple perspectives may allow for more accurate evaluation of post-intervention changes. Combining subjective and objective indicators may also enhance the effectiveness of the nutrition management cycle, including planning, implementation, evaluation, and improvement. In addition, longer-term and repeated nutrition education interventions may contribute to improvements in body composition and lipid-related outcomes.

The findings of this review further suggest that, in addition to alignment between intervention objectives and outcome measures, the role of professionals delivering nutrition education is critical to enhancing effectiveness. Interventions led by sports nutrition specialists reported relatively consistent improvements in health and nutritional status. Accordingly, future efforts should focus on strengthening dietitians’ competencies in behavior change theories and evaluation methods, as well as establishing nutrition education systems in athletic settings based on multidisciplinary collaboration among dietitians, coaches, trainers, and medical staff.

### Limitations

This review has several limitations that should be considered when interpreting the effects of nutrition education interventions on athletes’ health and nutritional status. First, the included studies were not limited to those that explicitly aimed to improve health or nutritional status as primary outcomes, and some studies did not clearly state intervention objectives, making rigorous evaluation of effectiveness difficult. Second, there was substantial heterogeneity in intervention types and durations, and particularly in short-term interventions, it is difficult to evaluate improvements in health and nutritional status that result from behavioral change. Future studies employing longer intervention periods are warranted. In addition, the included studies covered athletes across a wide age range, including adolescent, collegiate, and adult athletes. Because adolescence is a period of growth and development, nutrition education interventions targeting younger athletes may place greater emphasis on promoting healthy eating behaviors and nutritional balance rather than improving long-term physiological health outcomes. Differences in developmental stage, sporting environment, access to nutrition and medical support, and feasibility of physiological or biochemical assessments may have influenced both intervention objectives and outcome selection, which may have contributed to variability in health-related outcome findings across studies. Therefore, caution is warranted when interpreting the consistency of health-related outcomes across these populations.

Third, although this review was conducted using a rigorous methodology, limitations in search strategies and database selection may have resulted in the omission of some relevant studies. Fourth, the methodological quality of several included studies was suboptimal. Many studies employed single-group pre–post designs, and few randomized controlled trials with appropriate control groups were identified. Even among RCTs, reporting of allocation procedures and blinding was often insufficient, suggesting a high risk of bias. These findings highlight the need for improved study designs in future nutrition education intervention research targeting athletes.

Despite the limited strength of the evidence, this review provides valuable insights into the importance of designing and evaluating nutrition education interventions for athletes from a nutrition management perspective.

## Conclusions

This systematic review demonstrated that, in nutrition education intervention studies targeting athletes, objective indicators of health and nutritional status—such as body weight, body composition, and hydration status—have been predominantly used, whereas studies evaluating subjective health status remain limited. Nutrition education interventions may be effective for intermediate outcomes, including nutrition knowledge, dietary behaviors, and self-efficacy; however, evidence for effectiveness in anthropometric/body composition and biochemical outcomes (e.g., body weight, body composition, and blood biomarkers) remains inconclusive. In addition, studies in which intervention objectives, educational content, and outcome measures were well aligned tended to report improvements in outcomes. These findings suggest that, to appropriately evaluate the effectiveness of nutrition education interventions, the selection of outcome measures aligned with intervention objectives and a staged evaluation from intermediate to final outcomes are essential. Repeated nutrition education interventions may contribute to sustainable behavioral and health-related changes in athletes.

## Supplementary Information

Supplementary Material 1.

Supplementary Material 2.

## Data Availability

All data generated or analyzed during this systematic review are included in this published article and its supplementary materials.

## Abbreviations

BMI: Body mass index
EAT-26: Eating Attitudes Test-26
EA: Energy availability
ESQ: Environmental Symptoms Questionnaire
GENIE: Guide for Effective Nutrition Interventions and Education
IOC: International Olympic Committee
KAP: Knowledge–Attitude–Practice
LEA: Low energy availability
NHLBI: National Heart, Lung, and Blood Institute
NRCT: Non-randomized controlled trial
PRISMA: Preferred Reporting Items for Systematic Reviews and Meta-Analyses
QOL: Quality of life
RCT: Randomized controlled trial
