# Supplementary material for: Outcome indicators and effectiveness of nutrition education for athletes: a systematic review
Source: BMC Sports Sci Med Rehabil. 2026 Jul 21;18:501. doi: 10.1186/s13102-026-01900-9 (PMC13673610; doi:10.1186/s13102-026-01900-9)
Supplement: Supplementary file 1 [file 13102_2026_1900_MOESM1_ESM.pdf]

A Systematic Review of the Effectiveness of Nutrition Education Interventions to Maintain and Improve Health and Nutritional Status for Athletes
**PUBMED/MEDLINE**
Athletes[Mesh]
AND
("education intervention" OR "nutritional intervention" OR "nutrition education" OR "diet education" OR "nutritional support" OR "nutritional program")
AND
("Health Status" OR "Nutritional Status" OR "Nutrition Assessment" OR Condition OR performance)
**Cochrane Library**
(Athletes OR sport)
AND
("education intervention" OR "nutritional intervention" OR "nutrition education" OR "diet education" OR "nutritional support" OR "nutritional program")
AND
("Health Status" OR "Nutritional Status" OR "Nutrition Assessment" OR Condition OR performance)
**Web of Science**
(Athletes OR sport)
AND
("education intervention" OR "nutritional intervention" OR "nutrition education" OR "diet education" OR "nutritional support" OR "nutritional program")
AND
("Health Status" OR "Nutritional Status" OR "Nutrition Assessment" OR Condition OR performance)
**SPORTDiscus**
（Athletes OR sport）
AND
("education intervention" OR "nutritional intervention" OR "nutrition education" OR "diet education" OR "nutritional support" OR "nutritional program")
AND
("Health Status" OR "Nutritional Status" OR "Nutrition Assessment" OR Condition OR performance)

**Eric**
（Athletes OR sport）
AND
("education intervention" OR "nutritional intervention" OR "nutrition education" OR "diet education" OR "nutritional support" OR "nutritional program")
AND
("Health Status" OR "Nutritional Status" OR "Nutrition Assessment" OR Condition OR performance)
**Embase（ELSEVIER)**
（Athletes OR sport）
AND
("education intervention" OR "nutritional intervention" OR "nutrition education" OR "diet education" OR "nutritional support" OR "nutritional program")
AND
("Health Status" OR "Nutritional Status" OR "Nutrition Assessment" OR Condition OR performance)
**Science Direct**
①（Athletes OR sport）
AND
"education intervention"
AND
("Health Status" OR "Nutritional Status" OR "Nutrition Assessment" OR Condition OR performance)
②（Athletes OR sport）
AND
"nutritional intervention"
AND
("Health Status" OR "Nutritional Status" OR "Nutrition Assessment" OR Condition OR performance)
③（Athletes OR sport）
AND
"nutrition education"

AND
("Health Status" OR "Nutritional Status" OR "Nutrition Assessment" OR Condition OR performance)
④（Athletes OR sport）
AND
"diet education"
AND
("Health Status" OR "Nutritional Status" OR "Nutrition Assessment" OR Condition OR performance)
⑤（Athletes OR sport）
AND
"nutritional support"
AND
("Health Status" OR "Nutritional Status" OR "Nutrition Assessment" OR Condition OR performance)
⑥（Athletes OR sport）
AND
"nutritional program"
AND
("Health Status" OR "Nutritional Status" OR "Nutrition Assessment" OR Condition OR performance)
