# Supplementary material for: Outcome indicators and effectiveness of nutrition education for athletes: a systematic review
Source: BMC Sports Sci Med Rehabil. 2026 Jul 21;18:501. doi: 10.1186/s13102-026-01900-9 (PMC13673610; doi:10.1186/s13102-026-01900-9)
Supplement: Supplementary file 2 [file 13102_2026_1900_MOESM2_ESM.pdf]

PRISMA 2020 Checklist

Section and Topic	Item #	Checklist item	Location where item is reported
**TITLE**			
Title	1	Identify the report as a systematic review.	P1<br>Title: Outcome Indicators and Effectiveness of Nutrition Education for Athletes: A Systematic Review
**ABSTRACT**			
Abstract	2	See the PRISMA 2020 for Abstracts checklist.	P3-4<br>Abstract（Background / Methods / Results / Conclusions）
**INTRODUCTION**			
Rationale	3	Describe the rationale for the review in the context of existing knowledge.	P5<br>Background<br>(IOC guidelines, RED-S, previous systematic reviews, and the discussion on the gap between knowledge and final outcomes)
Objectives	4	Provide an explicit statement of the objective(s) or question(s) the review addresses.	P6 L96-100<br>background<br>The objectives of this systematic review were as follows: (1) to summarize health and nutritional status outcome indicators assessed before and after nutrition education interventions targeting athletes; and (2) to examine the effectiveness of these interventions in relation to the consistency of outcome changes and the relationships among intervention objectives, educational content, and intermediate outcomes.
**METHODS**			
Eligibility criteria	5	Specify the inclusion and exclusion criteria for the review and how studies were grouped for the syntheses.	P7 L121-P8 141<br>Eligibility criteria
Information sources	6	Specify all databases, registers, websites, organisations, reference lists and other sources searched or consulted to identify studies. Specify the date when each source was last searched or consulted.	P6 L102-105<br>Methods → Study design and registration（7 databases, search dates）
Search strategy	7	Present the full search strategies for all databases, registers and websites, including any filters and limits used.	P7 L108-112<br>Methods → Study design and registration<br>（Explicit keyword search strategy）<br>The full search strategies for all databases are provided in Supplementary File 1.
Selection process	8	Specify the methods used to decide whether a study met the inclusion criteria of the review, including how many reviewers screened each record and each report retrieved, whether they worked	P8 L142-P9 L151

PRISMA 2020 Checklist

Section and Topic	Item #	Checklist item	Location where item is reported
		independently, and if applicable, details of automation tools used in the process.	Methods → Study selection and data extraction (independent assessments by TW, NE, NS, and IT)
Data collection process	9	Specify the methods used to collect data from reports, including how many reviewers collected data from each report, whether they worked independently, any processes for obtaining or confirming data from study investigators, and if applicable, details of automation tools used in the process.	P8 L142-P9 L151<br>Methods → Study selection and data extraction<br>two authors (TW and NS)
Data items	10a	List and define all outcomes for which data were sought. Specify whether all results that were compatible with each outcome domain in each study were sought (e.g. for all measures, time points, analyses), and if not, the methods used to decide which results to collect.	P7 L121-P9 161<br>Methods → Eligibility criteria + Study selection and data extraction（final vs intermediate outcomes）
	10b	List and define all other variables for which data were sought (e.g. participant and intervention characteristics, funding sources). Describe any assumptions made about any missing or unclear information.	P8 142-P9 161<br>Methods → Study selection and data extraction<br>（country, age, sex, sport, objectives, format etc）
Study risk of bias assessment	11	Specify the methods used to assess risk of bias in the included studies, including details of the tool(s) used, how many reviewers assessed each study and whether they worked independently, and if applicable, details of automation tools used in the process.	P9 162-P10 173<br>Methods → Quality assessment（NHLBI tools）
Effect measures	12	Specify for each outcome the effect measure(s) (e.g. risk ratio, mean difference) used in the synthesis or presentation of results.	Methods<br>The Results are described as a qualitative synthesis. Because of heterogeneity in outcome measures and study designs, no quantitative effect measures (e.g., mean difference or risk ratio) were calculated, and results were synthesized narratively based on pre–post changes and consistency of improvement.
Synthesis methods	13a	Describe the processes used to decide which studies were eligible for each synthesis (e.g. tabulating the study intervention characteristics and comparing against the planned groups for each synthesis (item #5)).	P9 L157-161, 148-151<br>Methods<br>「Study designs were categorized as RCTs, NRCTs, or single-group pre–post intervention studies. Studies described by the authors as RCTs or NRCTs were reclassified as single-group pre–post intervention studies if the reported results were limited to within-group pre–post comparisons.」<br>「Outcomes were classified based on the Knowledge–Attitude–Practice (KAP) model into intermediate outcomes (knowledge, attitudes, self-efficacy, and dietary behaviors) and final outcomes (body composition, blood biomarkers, hydration status, and subjective health status).
	13b	Describe any methods required to prepare the data for presentation or synthesis, such as handling of missing summary statistics, or data conversions.	Methods → outcome classification, reclassification of RCT/NRCT
	13c	Describe any methods used to tabulate or visually display results of individual studies and syntheses.	Table 1, Table 2, Table 3, Table 4

PRISMA 2020 Checklist

Section and Topic	Item #	Checklist item	Location where item is reported
	13d	Describe any methods used to synthesize results and provide a rationale for the choice(s). If meta-analysis was performed, describe the model(s), method(s) to identify the presence and extent of statistical heterogeneity, and software package(s) used.	Results & Discussion（narrative synthesis based on consistency & alignment）
	13e	Describe any methods used to explore possible causes of heterogeneity among study results (e.g. subgroup analysis, meta-regression).	Discussion（differences by objective, hydration vs body comp etc）
	13f	Describe any sensitivity analyses conducted to assess robustness of the synthesized results.	Not conducted（no sensitivity analysis described）
Reporting bias assessment	14	Describe any methods used to assess risk of bias due to missing results in a synthesis (arising from reporting biases).	Limitations（publication bias and database limitations）. No formal assessment of reporting bias (e.g., funnel plots or statistical tests) was performed because a quantitative synthesis was not conducted.
Certainty assessment	15	Describe any methods used to assess certainty (or confidence) in the body of evidence for an outcome.	Limitations + Discussion（methodological weakness, heterogeneity, inconclusive evidence）
**RESULTS**			
Study selection	16a	Describe the results of the search and selection process, from the number of records identified in the search to the number of studies included in the review, ideally using a flow diagram.	P10 L184-P11 L194<br>Results<br>Study selection + Figure 1
	16b	Cite studies that might appear to meet the inclusion criteria, but which were excluded, and explain why they were excluded.	P10 L188-189<br>Results<br>Study selection（50 excluded after full-text）
Study characteristics	17	Cite each included study and present its characteristics.	P12-15<br>Results<br>Participant characteristics + Table 1
Risk of bias in studies	18	Present assessments of risk of bias for each included study.	P21-23<br>Results<br>Quality assessment of study design + Tables 3– 4
Results of individual studies	19	For all outcomes, present, for each study: (a) summary statistics for each group (where appropriate) and (b) an effect estimate and its precision (e.g. confidence/credible interval), ideally using structured tables or plots.	Table 1（health/nutrition outcomes）
Results of syntheses	20a	For each synthesis, briefly summarise the characteristics and risk of bias among contributing studies.	P16-17<br>Results<br>Intervention characteristics and effectiveness
	20b	Present results of all statistical syntheses conducted. If meta-analysis was done, present for each the summary estimate and its precision (e.g. confidence/credible interval) and measures of statistical heterogeneity. If comparing groups, describe the direction of the effect.	P16-17<br>Results<br>Intervention characteristics and effectiveness

PRISMA 2020 Checklist

Section and Topic	Item #	Checklist item	Location where item is reported
			（dehydration, weight, LEA etc）
	20c	Present results of all investigations of possible causes of heterogeneity among study results.	P16-17<br>Results<br>Intervention characteristics and effectiveness （objective/content alignment, inconsistent weight outcomes）
	20d	Present results of all sensitivity analyses conducted to assess the robustness of the synthesized results.	Not conducted
Reporting biases	21	Present assessments of risk of bias due to missing results (arising from reporting biases) for each synthesis assessed.	Limitations<br>No formal assessment of reporting bias (e.g., funnel plots or statistical tests) was performed because a quantitative synthesis was not conducted.
Certainty of evidence	22	Present assessments of certainty (or confidence) in the body of evidence for each outcome assessed.	Limitations + Discussion
**DISCUSSION**			
Discussion	23a	Provide a general interpretation of the results in the context of other evidence.	Discussion (Overall interpretation)
	23b	Discuss any limitations of the evidence included in the review.	Limitations（study design, heterogeneity）
	23c	Discuss any limitations of the review processes used.	Limitations（search strategy, databases）
	23d	Discuss implications of the results for practice, policy, and future research.	Discussion（nutrition management framework, professional roles, future research）
**OTHER INFORMATION**			
Registration and protocol	24a	Provide registration information for the review, including register name and registration number, or state that the review was not registered.	Methods → PROSPERO (CRD42023417419)
	24b	Indicate where the review protocol can be accessed, or state that a protocol was not prepared.	Not separately published（Registration only in the Methods section）
	24c	Describe and explain any amendments to information provided at registration or in the protocol.	Updated search March 2025
Support	25	Describe sources of financial or non-financial support for the review, and the role of the funders or sponsors in the review.	Declarations → Funding
Competing interests	26	Declare any competing interests of review authors.	Declarations → Competing interests
Availability of data, code and other materials	27	Report which of the following are publicly available and where they can be found: template data collection forms; data extracted from included studies; data used for all analyses; analytic code; any other materials used in the review.	Declarations → Availability of Data and Materials

From: Page MJ, McKenzie JE, Bossuyt PM, Boutron I, Hoffmann TC, Mulrow CD, et al. The PRISMA 2020 statement: an updated guideline for reporting systematic reviews. BMJ 2021;372:n71. doi: 10.1136/bmj.n71
